# LASSO-derived model for the prediction of lean-non-alcoholic fatty liver disease in examinees attending a routine health check-up

**DOI:** 10.1080/07853890.2024.2317348

**Published:** 2024-02-16

**Authors:** Chiao-Lin Hsu, Pin-Chieh Wu, Fu-Zong Wu, Hsien-Chung Yu

**Affiliations:** a Health Management Center, Kaohsiung Veterans General Hospital, Kaohsiung, Taiwan; b Center for Geriatrics and Gerontology, Kaohsiung Veterans General Hospital, Kaohsiung, Taiwan; c Department of Radiology, Kaohsiung Veterans General Hospital, Kaohsiung, Taiwan; d Faculty of Medicine, School of Medicine, National Yang-Ming University, Taipei, Taiwan; e Department of Medical Imaging and Radiology, Shu-Zen Junior College of Medicine and Management, Kaohsiung, Taiwan; f Department of Internal Medicine of Kaohsiung Veterans General Hospital, Kaohsiung, Taiwan

**Keywords:** Lean, non-alcoholic fatty liver disease, diagnosis, prediction model, nomogram

## Abstract

**Background:**

Lean individuals with non-alcohol fatty liver disease (NAFLD) often have normal body size but abnormal visceral fat. Therefore, an alternative to body mass index should be considered for prediction of lean-NAFLD. This study aimed to use representative visceral fat links with other laboratory parameters using the least absolute shrinkage and selection operator (LASSO) method to construct a predictive model for lean-NAFLD.

**Methods:**

This retrospective cross-sectional analysis enrolled 2325 subjects with BMI < 24 kg/m^2^ from medical records of 51,271 examinees who underwent a routine health check-up. They were randomly divided into training and validation cohorts at a ratio of 1:1. The LASSO-derived prediction model used LASSO regression to select 23 clinical and laboratory factors. The discrimination and calibration abilities were evaluated using the Hosmer–Lemeshow test and calibration curves. The performance of the LASSO model was compared with the fatty liver index (FLI) model.

**Results:**

The LASSO-derived model included four variables—visceral fat, triglyceride levels, HDL-C-C levels, and waist hip ratio—and demonstrated superior performance in predicting lean-NAFLD with high discriminatory ability (AUC, 0.8416; 95% CI: 0.811–0.872) that was comparable with the FLI model. Using a cut-off of 0.1484, moderate sensitivity (75.69%) and specificity (79.86%), as well as high negative predictive value (95.9%), were achieved in the LASSO model. In addition, with normal WC subgroup analysis, the LASSO model exhibits a trend of higher accuracy compared to FLI (cut-off 15.45).

**Conclusions:**

We developed a LASSO-derived predictive model with the potential for use as an alternative tool for predicting lean-NAFLD in clinical settings.

## KEY MESSAGES

1. Researchers developed a model to predict a type of liver disease called non-alcoholic fatty liver disease (NAFLD) in lean individuals.
2. The model accurately detects NAFLD in lean individuals using factors like visceral fat, triglyceride levels, and waist-to-hip ratio, aiding in identifying the disease in normal-weight people with abnormal fat distribution

## 1. Introduction

Non-alcoholic fatty liver disease (NAFLD) is a prevalent liver disease worldwide with a global prevalence of 25.24% and an estimated prevalence of 27.4% in Asia according to one meta-analysis [1]. With the increasing number of people with obesity and an aging global population, the prevalence of NAFLD is expected to rise. However, the disease is not solely caused by increased body weight (BW), as the prevalence of NAFLD is higher in younger populations, even those with a normal or low weight. According to a review, the worldwide prevalence of NAFLD is 10–20% in Caucasians and 11–53% in Asian populations. Shi et al. reported a gradual increase in the prevalence of lean-NAFLD from 5.6% to 12.6% after 2000 [2–4].

NAFLD is a serious liver disease associated with extrahepatic conditions, and advanced fibrosis resulting from NAFLD can lead to liver-related mortality and hepatocellular carcinoma [5]. Although lean-NAFLD patients are initially considered to have a less severe form of the disease, recent studies have demonstrated a similar long-term prognosis in lean-NAFLD patients and those with obesity [6–8]. A large retrospective cohort study also indicated that a lean status can be associated with liver-related adverse events and overall mortality [9].

The fatty liver index (FLI), which considers body mass index (BMI), waist circumference (WC), triglyceride levels, and gamma-glutamyl transferase (GGT), has been widely used to predict NAFLD [10–13] and has moderate-to-high predictive power for fatty liver in lean populations [14]. Although BMI is frequently used to categorize obesity, it is not a sufficient indicator of central obesity. Moreover, considering that lean individuals often have normal body size, but may have abnormalities in visceral fat, BMI cannot be used as the sole indicator to assess the severity and prognosis of NAFLD. One review article suggested visceral adiposity may be a critical risk factor for lean-NAFLD [15]. Furthermore, previous studies have shown that compared to BMI and WC, waist-hip ratio (WHR) is a superior predictor of visceral fat, which is strongly linked to fatty liver [16,17]. Zheng et al. [18] also reported a strong association between WHR and NAFLD, with WHR being an indicator of hepatic steatosis risk, even in adolescents [19,20]. Therefore, measuring and monitoring visceral adiposity may provide a better understanding of the relationship between body composition and health outcomes.

This study aimed to use representative visceral fat links with other laboratory parameters and the least absolute shrinkage and selection operator (LASSO) method to construct a predictive model for lean-NAFLD that has better clinical index significance than the FLI for the lean population.

## 2. Methods

### 2.1. Subjects

**Figure 1.**
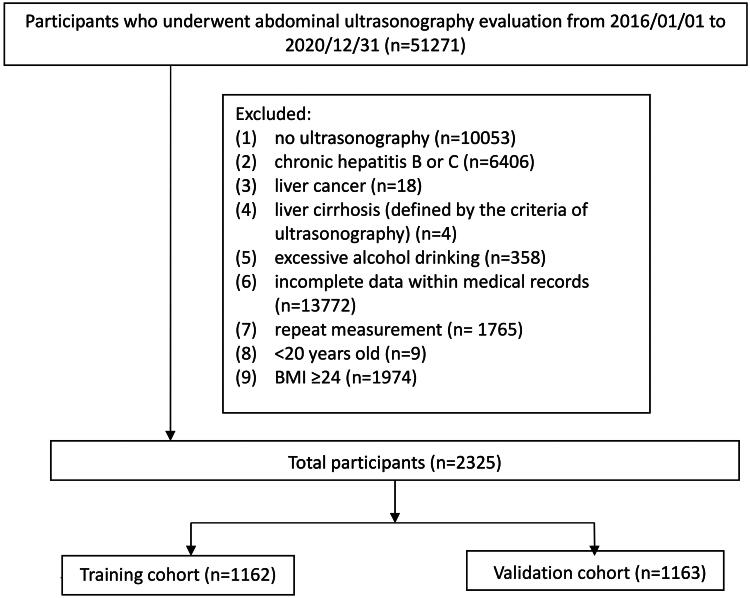
Flow chart of the study. BMI: body mass index

This retrospective cross-sectional study was conducted using the de-identified medical records of 51,271 examinees who underwent routine health checkups at the Health Examination Center of Kaohsiung Veterans General Hospital between January 1, 2016, and December 31, 2020. The flow chart of the study is shown in Figure 1. We excluded patients whose records indicated at least one of the following: (1) significant consumption of alcohol, defined as 0.20 g/d for men and 0.10 g/d for women, according to the National Health and Nutrition Examination Survey III criteria [21]; (2) liver cirrhosis (defined by ultrasonographic criteria); (3) chronic hepatitis B or C (defined by history, serum hepatitis B surface antigen, and anti-hepatitis C antibody); (4) liver cancer; and (5) lack of ultrasonographic examination in the health checkup data, repeat measurements, or incomplete data. Lastly, 2325 examinees with a BMI <24 kg/m^2^ were enrolled in the lean patient population. NAFLD was defined as an ultrasonographic diagnosis of a fatty liver. This study was approved by the Institutional Review Board of the Kaohsiung Veterans General Hospital (no. 21-CT2-08(210125-1)). Written consent from the study patients was not necessary as the dataset consisted of de-identified data for research purposes.

### 2.2. Measurements

The weight, height, and body fat mass of all examinees were measured using an electric impedance method analyzer (XSCAN PLUS II; Jawon Medical, Gyeongsan-si, South Korea), with the patients wearing minimal clothes and no socks. BMI was calculated as weight (kg) divided by height (m) squared. Well-trained examiners used a non-stretchable tape measure without exerting pressure on the body surface to measure the WC of all examinees at the umbilical level. A trained examiner measured all anthropometric indices. Abdominal ultrasonographic examinations to determine hepatic fat infiltration were performed by the same five experienced ultrasonographic technicians using a GE LOGIQ E9 ultrasound machine (GE Healthcare, Chalfont St. Giles, United Kingdom). The measurements were verified by five experienced senior radiologists, each with 10 years of experience. The criteria for the diagnosis and severity of fatty liver on ultrasonography were established according to the practice guidelines of the American Gastroenterology Association.

### 2.3. Serological and biochemical markers

Hematological indicators were measured using a hematology analyzer (UniCel DxH 800; Beckman Coulter, Brea, CA, USA). Biochemical indicators included fasting plasma glucose, hemoglobin A1c (HbA1c), serum uric acid, total cholesterol, triglyceride levels, high density lipoprotein-cholesterol (HDL-C); low density lipoprotein-cholesterol(LDL-C), aspartate aminotransferase, alanine aminotransferase, GGT, and alkaline phosphatase levels. All serum biochemical markers were measured using a Hitachi 7600 Automatic Biochemical Analyzer (Hitachi, Tokyo, Japan). Serum HbA1c levels were analyzed using a Premier Hb9210 HbA1c Analyzer (Bray, Ireland, Kansas City, MO, USA). Hepatitis B surface antigen was measured using radioimmunoassay kits (Ausria II-125; Abbott Laboratories, North Chicago, IL, USA) and anti-hepatitis C antibody was measured using a microparticle enzyme immunoassay (Ax SYM HCV III; Abbott Laboratories). All blood samples were obtained after an 8-h overnight fast.

The overall dataset (*n* = 2325) was randomly divided into two groups: the training dataset (*n* = 1162) and validation cohort (*n* = 1163).

### 2.4. LASSO-derived prediction model

We analyzed 23 features, including age, sex, BMI, WC, BW, body fat, WHR, history of hypertension (HTN) and diabetes mellitus (DM), fasting glucose, exercise frequency, alcohol drinking frequency, platelet count, alkaline phosphatase, HbA1c, cholesterol, HDL-C, LDL-C, triglyceride levels, uric acid, muscle mass, visceral fat, and total cholesterol/HDL-C ratio. LASSO regression was used to construct a new prediction model with an optimal lambda value that minimized the cross-validation error and to compare its prediction accuracy and discriminatory ability with that of the FLI model. Finally, we extracted 4 features (visceral fat, triglyceride, HDL-C, and waist and hip ratio) through LASSO regression to construct the new prediction model with the optimal value of lambda that minimizes the cross-validation error and compares its prediction accuracy and discriminatory ability with FLI prediction model.

### 2.5. FLI model

Bedogni et al. [22] originally developed the FLI which could accurately distinguish patients with NAFLD. Yang et al. [23] also suggested that the FLI was a reliable non-invasive predictor of NAFLD in both Asian and Western populations.

The FLI was calculated using the following formula: FLI = (e0.953 ∗ log^e^ (triglycerides) + 0.139 ∗ BMI + 0.718 ∗ log^e^ (GGT) + 0.053 ∗ WC − 15.745)/(1 + e0.953 ∗ log^e^ (triglycerides) + 0.139 ∗ BMI + 0.718 ∗ log^e^ (GGT) +0.053 ∗ WC − 15.745) ∗ 100.

### 2.6. Statistical analysis

Student’s t-tests were used to compare the continuous variables among the clinical and demographic characteristics of the subjects in the training and validation groups. Chi-squared and Fisher’s exact tests were used for categorical variables. The primary study outcome was the development of a LASSO-derived prediction model (optimal lambda selection) for lean-NAFLD in the Asian population.

Multiple logistic regression models were applied to estimate the odds ratios (OR) and 95% CIs. We evaluated and compared the discriminatory ability of the predictive models using the C-statistic (AUC), Akaike information criterion (AIC), and Bayesian information criterion (BIC). Models with higher C-statistics and lower AIC/BIC values were regarded as having a higher discriminatory ability. C-statistic values ranged from 0.5 (no ability to discriminate) to 1.0 (full ability to discriminate). The Hosmer–Lemeshow goodness-of-fit statistic was used for calibration.

A model was established according to the LASSO-derived parameters in the training cohort. Statistical significance for all tests was set at *p* < .05. All statistical analyses were performed using SPSS for Windows (version 22.0; SPSS Inc., Chicago, IL, USA) and Stata version 13.0 (Stata Corp, College Station, TX, USA).

**Figure 2.**
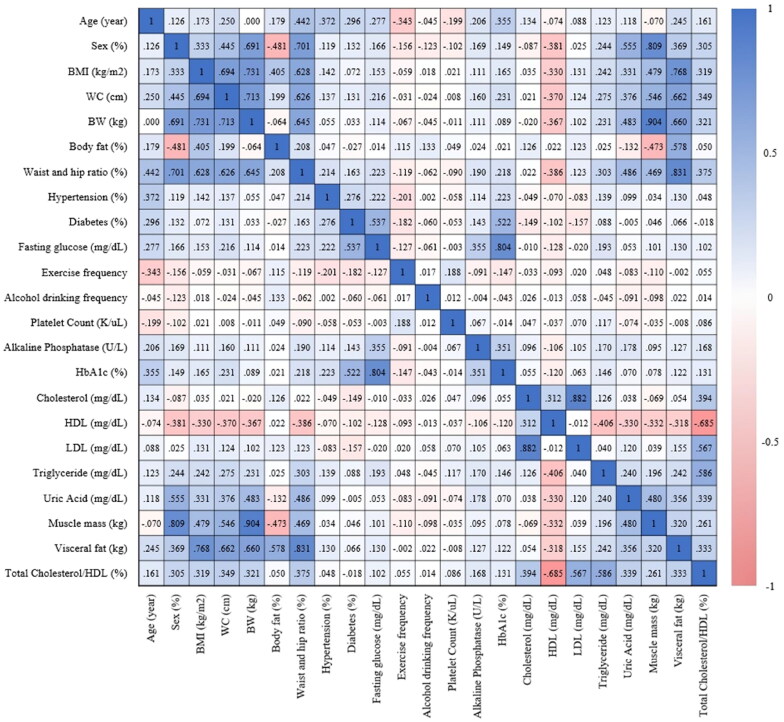
This heatmap visually represents the correlation matrix of 23 features, including age, sex, BMI, WC, BW, body fat, WHR, history of hypertension (HTN) and diabetes mellitus (DM), fasting glucose, exercise frequency, alcohol drinking frequency, platelet count, alkaline phosphatase, HbA1c, cholesterol, HDL-C, LDL-C, triglyceride levels, uric acid, muscle mass, visceral fat, and total cholesterol/HDL-C ratio. The color spectrum in the heatmap spans from deep blue, denoting positive correlations, to deep red, indicating negative correlations.

The statistical approach employed a combination of the LASSO algorithm and Principal Component Analysis (PCA) to identify key features associated with the main study outcome. Emphasis was placed on identifying principal components that collectively accounted for 80% of all features. PCA played a pivotal role in addressing multicollinearity by transforming highly correlated variables into a set of independent variables. This process of feature selection and dimensionality reduction was visually presented using a heatmap (refer to Figure 2). To further enhance the analysis, the LASSO algorithm was subsequently utilized to determine the optimal parameters for model development and the establishment of a nomogram. In summary, both the LASSO algorithm and PCA were instrumental in identifying crucial features related to lean-NAFLD. Principal components, contributing to 80% of overall features, were selected, and PCA was additionally employed to further refine for training the predictive model, preventing overfitting due to redundant features. Ultimately, four parameters were chosen to construct the predictive models.

## 3. Results

### 3.1. Study population characteristics

Of the 2325 subjects in our study, 852 were men. The prevalence of lean-NAFLD in the study cohort was 12.9% (301 among 2325 subjects). The subjects were randomly assigned to the training or validation groups at a ratio of 1:1. In total, 1162 and 1163 subjects were included in the training and validation groups, respectively. Table 1 shows the basic clinical characteristics for the training (mean age, 48.32 ± 12.33 years, 36.3% men) and the validation cohorts (mean age, 47.83 ± 12.50 years, 37.0% men). The number of patients with lean-NAFLD in the training and validation groups was 154 (13.3%) and 147 (12.6%) (*p* = .66), respectively. There were no significant differences in basic clinical characteristics between the training and validation groups, except for hypertension history (139 [12%] vs. 107 [9.2%]; *p* = .03) and serum alkaline phosphatase levels (62.59 ± 22.51 vs. 60.70 ± 18.78; *p* = .032).

**Table 1.** Clinical characteristics of the training and validation sets.

	Training cohort (*n* = 1162)	Validation cohort (*n* = 1163)	*p*-value
Age (year)	48.32 ± 12.33	47.83 ± 12.50	.343
Sex (%)			.742
female	740 (63.7%)	733 (63%)	
male	422 (36.3%)	430 (37%)	
BMI (kg/m2)	21.22 ± 1.85	21.21 ± 1.91	.903
WC (cm)	78.04 ± 7.00.	77.59 ± 7.10	.125
BW (kg)	57.76 ± 8.45	58.00 ± 8.50	.487
Body fat (%)	21.64 ± 5.26	21.52 ± 5.38	.598
Waist and hip ratio (%)	0.80 ± 0.08	0.800 ± 0.08	.915
Hypertension (%)	139 (12%)	107 (9.2%)	.030*
Diabetes (%)	66 (5.7%)	54 (4.6%)	.259
Exercise frequency			.932
≥ 5 times/ week	137 (11.8%)	143 (12.3%)	
3–5 times/week	288 (24.8%)	286 (24.6%)	
<3 times/week	737 (63.4%)	734 (63.1%)	
Alcohol drinking frequency			.785
≥ 3 times/ week	60 (5.2%)	63 (5.4%)	
<3 times/week	1102 (94.8%)	1100 (94.6%)	
Fasting glucose (mg/dL)	91.14 ± 18.77	89.97 ± 19.42	.137
HbA1c (%)	5.77 ± 0.67	5.73 ± 0.72	.138
Cholesterol (mg/dL)	198.86 ± 37.48	197.22 ± 37.89	.293
HDL (mg/dL)	57.06 ± 14.24	57.44 ± 14.26	.525
LDL (mg/dL)	121.94 ± 33.34	120.56 ± 33.33	.318
Total Cholesterol/HDL (%)	3.66 ± 1.04	3.62 ± 1.06	.271
Triglyceride (mg/dL)	101.44 ± 70.42	98.41 ± 66.07	.285
Alkaline Phosphatase (U/L)	62.59 ± 22.51	60.70 ± 18.78	.032*
Platelet Count (K/uL)	248.94 ± 61.47	246.00 ± 57.79	.235
Muscle mass (kg)	42.00 ± 7.19	42.23 ± 7.24	.434
Visceral fat (kg)	1.34 ± 0.52	1.34 ± 0.53	.874
Fatty liver (%)	154 (13.3%)	147 (12.6%)	.660

Abbreviation: BMI: Body Mass Index; WC: Waist circumference; BW: Body weight; HbA1c: hemoglobin A1c; HDL-C: high density lipoprotein-cholesterol; LDL-C: low density lipoprotein-cholesterol.

Table 2 shows demographic data comparisons between individuals with and without NAFLD among training set. The data includes sex, HTN, DM, exercise frequency, alcohol drinking frequency, age, BMI, WC, BW, WHR, fasting glucose, HbA1c, alkaline phosphatase, total cholesterol, HDL-C, LDL-C, total cholesterol/HDL-C ratio, triglyceride levels, platelet count, muscle mass, and visceral fat. The results indicate that the values for all variables were significantly different between the two groups, except for exercise frequency and alcohol drinking frequency.

**Table 2.** Demographic data comparison based on non-alcoholic fatty liver status, stratified from training set.

		no fatty liver (*n* = 1008)	fatty liver (*n* = 154)	*p*-value
Age(year)		47.68 ± 12.46	52.51 ± 10.58	<.005
Sex (%)	female	680 (67%)	60 (39%)	<.005
	male	328 (33%)	94 (61%)	
BMI (kg/m^2^)		21.04 ± 1.87	22.41 ± 1.18	<.005
WC (cm)		77.31 ± 6.92	82.79 ± 5.49	<.005
body weight (kg)		56.99 ± 8.27	62.81 ± 7.95	<.005
Body fat (%)		21.45 ± 5.33	22.86 ± 4.61	.032
Waist hip ratio		0.79 ± 0.08	0.86 ± 0.07	<.005
Hypertension	(%)	103 (10%)	36 (23%)	<.005
Diabetes mellitus	(%)	48 (5%)	18 (12%)	.001
Exercise frequency	≥ 5 times/ week	118 (12%)	19 (12%)	.957
	3–5 times/week	251 (25%)	37 (24%)	
	<3 times/week	639 (63%)	98 (64%)	
Alcohol drinking frequency	≥ 3 times/ week	53 (5%)	7 (5%)	.710
	<3 times/week	955 (95%)	147 (95%)	
Fasting glucose (mg/dL)		90.19 ± 18.17	97.39 ± 21.31	<.005
HbA1c (%)		5.73 ± 0.65	6.05 ± 0.73	< .005
Cholesterol (mg/dL)		198.95 ± 37.55	198.29 ± 37.16	.837
HDL-C (mg/dL)		58.67 ± 14.13	46.55 ± 9.81	<.005
LDL-C (mg/dL)		121.54 ± 33.40	124.62 ± 32.92	.286
Total Cholesterol/HDL-C (%)		3.55 ± 1.00	4.40 ± 1.05	<.005
Triglyceride (mg/dL)		92.23 ± 57.98	161.73 ± 106.24	<.005
Alkaline Phosphatase (U/L)		61.65 ± 22.96	68.67 ± 18.23	<.005
Platelet Count (K/uL)		247.48 ± 60.94	258.53 ± 64.25	.038
Muscle mass (kg)		41.53 ± 7.00	45.08 ± 7.64	<.005
Visceral fat (kg)		1.28 ± 0.51	1.73 ± 0.44	<.005

BMI: Body Mass Index; WC: Waist circumference; BW: Body weight; HbA1c: hemoglobin A1c; HDL-C: high density lipoprotein-cholesterol; LDL-C: low density lipoprotein-cholesterol.

### 3.2. LASSO-derived predictor for lean-NAFLD

In order to prevent overfitting, we utilized LASSO regression for parameter selection during model construction. This also addressed the problem of multicollinearity and excessive feature variables and identified the smallest sub-setting with the strongest interpretation effect and the most consistent variables. Herein, LASSO selection was performed *via* a 10-fold cross-validation for lean-NAFLD prediction. Ultimately, 23 variables were selected for the prediction model. The final four non-zero variables with optimal lambda in the LASSO model were visceral fat, triglyceride levels, HDL-C levels, and WHR. We conducted multivariate analyses of the training cohort to establish a predictive model for lean-NAFLD. Four of the original 23 variables were included in the predictive model. The LASSO-derived prediction model, including four selected variables, demonstrated superior performance (OR: 675.876; 95% CI, 230.373–1982.906; *p* < .001). (Table 3)

**Table 3.** Univariable and LASSO-derived multivariable logistic regression for predicting fatty liver in subjects with lean BMI.

	OR	CI	*p*-value
Univariate analysis			
Age (year)	1.035	1.025–1.045	<.001
Sex^a^ (%)	2.808	2.193–3.595	<.001
BMI (kg/m2)	1.768	1.606–1.946	<.001
WC (cm)	1.135	1.112–1.159	<.001
BW (kg)	1.078	1.063–1.094	<.001
Body fat (%)	1.063	1.038–1.089	<.001
Waist and hip ratio (%)	67023.428	12074.297–372041.533	<.001
Hypertension (%)	2.877	2.104–3.935	<.001
Diabetes (%)	4.046	2.720–6.017	<.001
Fasting glucose (mg/dL)	1.019	1.014–1.025	<.001
Exercise frequency	1.055	0.885–1.258	.548
Alcohol drinking frequency	0.860	0.514–1.440	.567
Platelet Count (K/uL)	1.003	1.001–1.005	.004
Alkaline Phosphatase (U/L)	1.017	1.010–1.023	<.001
HbA1c (%)	1.919	1.611–2.287	<.001
Cholesterol (mg/dL)	1.002	0.999–1.005	.295
HDL-C (mg/dL)	0.920	0.909–0.932	<.001
LDL-C (mg/dL)	1.005	1.002–1.009	.003
Triglyceride (mg/dL)	1.013	1.012–1.015	<.001
Uric Acid (mg/dL)	1.699	1.557–1.854	<.001
Muscle mass (kg)	1.058	1.041–1.075	<.001
Visceral fat (kg)	5.393	4.149–7.009	<.001
Total Cholesterol/HDL-C (%)	2.150	1.918–2.411	<.001
LASSO-derived model			
Prediction model	675.876	230.373–1982.906	<.001

Abbreviation: BMI: Body Mass Index; WC: Waist circumference; BW: Body weight; HbA1c: hemoglobin A1c; HDL-C: high density lipoprotein-cholesterol; LDL-C: low density lipoprotein-cholesterol.

^a^ Female sex: reference.

### 3.3. LASSO-derived model for nomogram development

**Figure 3.**
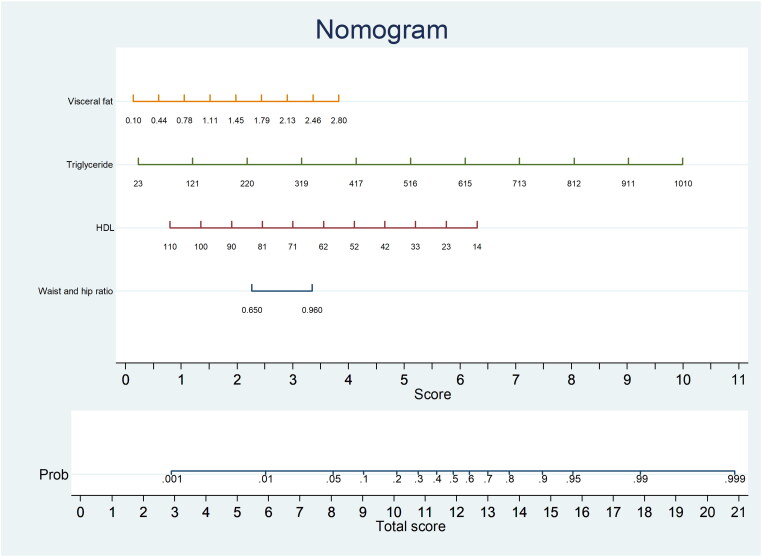
Nomogram for lean nonalcoholic fatty liver disease prediction.

The probability of lean-NAFLD in the study training cohort, according to the multivariable logistic regression model, included 4 potential predictive factors: visceral fat, triglyceride levels, HDL-C levels, and WHR. Each significant variable was assigned a score based on a point scale. A straight line was drawn to estimate the probability of lean-NAFLD at each time point by summing the total scores and locating them on a total point scale. For example, if the patient’s visceral fat was 1.45, it was first located on the relevant axis. Next, a straight line was drawn downward to the point axis (5th row, named ‘score’) to obtain the points based on visceral fat (2 points). Then, we repeated this course for triglyceride levels, HDL-C levels, and WHR. After that, we summed up all the points to obtain the ‘total score’ (the bottom row). Finally, a straight line was drawn upward from the 6th row to determine the probability of developing lean-NAFLD. That is, if a patient had visceral fat of 1.45 (2 points), triglyceride level of 220 (2 points), HDL-C level of 33 (5 points), and WHR of 0.96 (3.5 points), there was a 60% probability of lean-NAFLD (Figure 3).

### 3.4. Assessment of the performance of the LASSO-developed model and FLI model for the prediction of lean-NAFLD

Table 4 summarizes the discriminatory abilities and diagnostic performances of the two prediction models. The LASSO-derived model had moderate sensitivity (75.69) and specificity (79.86). The positive predictive value was 34.8 and the negative predictive value was 95.9. The LASSO-derived model had a higher specificity than the FLI model. Table 5 compares the discriminatory ability and diagnostic performance of the LASSO-derived model and the FLI model. The LASSO-derived model had high discriminatory ability (AUC, 0.8416; 95% CI: 0.811–0.872) and low AIC (709.610) and BIC (734.870). There was no significant difference between the LASSO-derived model and the FLI model (*p* = .470).

**Table 4.** Prediction performance of LASSO and FLI models, *n* = 1163.

Model	Cut point	Sensitivity	95% CI	Specificity	95% CI	+LR	95% CI	−LR	95% CI	+PV	95% CI	−PV	95% CI
LASSO	>0.1484	75.69	67.9–82.4	79.86	77.3–82.3	3.76	3.4–4.1	0.3	0.2–0.4	34.8	29.6–40.4	95.9	94.3–97.1
FLI	>15.45	76.19	68.5–82.8	78.35	75.7–80.8	3.52	3.2–3.9	0.3	0.2–0.4	33.7	28.7–39.1	95.8	94.2–97.0

LASSO: least absolute shrinkage, and selection operator; AIC: Akaike information criterion; BIC: Bayesian information criterion; AUC: area under the ROC curve; CI: confidence interval; LR: likelihood ratio; PV: predictive value.

**Table 5.** Prediction performance and comparison of LASSO and FLI models.

Model	AIC	BIC	AUC	95% CI	*p*-value*
LASSO	709.610	734.870	0.8416	0.811–0.872	.470
FLI	755.360	765.476	0.8322	0.801–0.863	

LASSO: least absolute shrinkage and selection operator; AIC: Akaike information criterion; BIC: Bayesian information criterion; AUC: area under the ROC curve; CI: confidence interval.

* LASSO vs. FLI model.

Given that individuals with normal BMI tend to have normal WC, we sought to compare the predictive performance of the LASSO model and the FLI for lean-NAFLD specifically in the context of normal WC. We constructed an additional table aimed to assess the efficacy of these models in predicting fatty liver when WC is within the normal range (males with WC < 90 cm and females with WC < 80 cm), particularly in populations where a significant proportion exhibit normal waist circumference despite having fatty liver. The cut-off value for FLI was 15.45, and 0.1484 for the LASSO model. FLI showed an accuracy of 0.790, with a sensitivity of 0.691 and a specificity of 0.803. The LASSO model showed a slightly higher accuracy of 0.797, with a higher sensitivity of 0.722, but a similar specificity of 0.807. Overall, both models demonstrated moderate accuracy in predicting lean-NAFLD. Based on the table provided, the LASSO model had a slightly higher accuracy than the FLI model in predicting fatty liver (Supplementary Table 1).

## 4. Discussion

Herein, we developed a LASSO-derived model for predicting lean-NAFLD that included four common risk factors. Although several studies [24–26] have previously evaluated the risk factors for predicting lean-NAFLD, to our knowledge, this is the first study to use a LASSO-derived model for lean-NAFLD prediction. In addition, we used electric impedance method analyzers to evaluate body composition and fat distribution, which provided a more thorough analysis of both total body fat and its distribution, enabling individuals to gain deeper insights into their overall health and make more knowledgeable choices regarding their diet and exercise habits. The major findings were as follows: first, high visceral fat, high triglyceride levels, low HDL-C levels, and high WHR were significantly correlated with lean-NAFLD; second, the four risk factors could be applied to form a suitable and useful nomogram to predict the probability of lean-NAFLD.

In most cases, patients with NAFLD are diagnosed incidentally, either during other medical imaging evaluations or routine annual physical checkups. However, body fat, height, and weight are frequently assessed in clinical settings. FLI is a formula that calculates the probability of NAFLD based on BMI, WC, GGT, and triglyceride levels, and it aids in the diagnosis of NAFLD without the need for invasive procedures such as liver biopsy [11]. To enhance the predictive capacity for lean-NAFLD, we conducted further investigations in response to the relatively low sensitivity (60%) observed in our previous study when utilizing a FLI cut-off point of 15 [14]. We postulated that the presence of unidentified factors, short follow-up period, and relatively small sample size may contribute to this limitation. To address this concern, we employed the LASSO model for predicting lean-NAFLD based on blood tests and anthropometric measurements. In the current study, we further compared FLI and the LASSO model as predictors of lean-NAFLD by setting the FLI cut-off point at 15.45. Both models demonstrated moderate accuracy in predicting lean-NAFLD. Given the importance of WHR and visceral fat in predicting fatty liver, we further compared the ability of FLI and LASSO model to predict NAFLD while holding WC constant. Both models demonstrated moderate accuracy in predicting lean-NAFLD, but the LASSO model was slightly more accurate than the FLI model in predicting fatty liver. Our findings suggest that the LASSO model may serve as an alternative tool for predicting lean-NAFLD, thus providing clinicians with more options for clinical decision-making.

We found that high visceral fat, high triglyceride levels, low HDL-C levels, and high WHR were associated with lean-NAFLD. Although both BMI and visceral adiposity demonstrate high accuracy and performance in identifying patients with lean-NAFLD, unlike BMI, which is a crude measure of body fat based on height and weight, visceral fat reflects the distribution of body fat and its impact on health. Measuring visceral fat using the LASSO model offers novel insights into body composition and its relationship with health outcomes. Research has shown that individuals with high levels of visceral fat are at an increased risk of chronic health conditions, even if their BMI falls within the ‘normal’ range [27].

Moreover, previous studies have reported a positive association between visceral fat—notably intra-abdominal fat—and NAFLD [28,29]; regarding NAFLD, visceral fat is more impactful than BMI [30]. One cohort study with a follow-up period of 4.4 years demonstrated that greater amounts of visceral fat were associated with a higher risk of developing NAFLD [31]. Herein, visceral fat played an important role in lean-NAFLD prediction, as implied by another predictor, WHR. WHR has been reported to be a superior indicator of abdominal obesity and visceral adiposity compared to WC. Increased visceral fat leads to increased insulin resistance with unrestrained lipolysis [32], and more free fatty acids and adipokines/cytokines are released into the portal circulation, which are key factors in NAFLD [33]. Individuals with normal BMI may have high visceral fat, and measuring BMI only underestimates the risk of NAFLD and overlooks this issue. Therefore, this study found that higher visceral fat was more influential than BMI in the lean-NAFLD population.

We also found that patients with lean-NAFLD and those with obesity share common metabolic abnormalities [3]. Low HDL-C levels [34,35] and high triglyceride [36] levels are closely associated with the development of NAFLD. Low HDL-C impairs the removal of cholesterol from liver cells, leading to its accumulation and contributing to fatty liver formation [37]. Elevated triglyceride levels result in increased triglyceride synthesis and reduced clearance, causing triglycerides to accumulate in liver cells and further promote fatty liver development. The interplay between low HDL-C and high triglyceride levels exacerbates the progression of fatty liver disease by impairing lipid metabolism and promoting lipid accumulation within hepatocytes.

Several studies have been conducted to construct prediction models for NAFLD. Cen et al. [38] employed six parameters, namely body fat mass, diastolic blood pressure, serum uric acid, fasting blood glucose, triglyceride levels, and alanine lipase levels, to predict NAFLD. These parameters align with the indicators used in our study, despite differences in the study population (overweight adults vs. normal or underweight adults). Several studies have also developed prediction models for lean-NAFLD. Su et al. [39] utilized a two-class neural network with 10 features, including BMI, WC, weight, age, blood pressure, serum triglyceride levels, serum HDL-C, glucose, and serum glutamic-pyruvic transaminase levels to predict NAFLD in individuals with a BMI <23 kg/m^2^. Liu et al. [40] developed a nomogram based on seven laboratory profiles, including triglyceride levels. Wang et al. [41] incorporated predictors such as triglycerides and HDL in their nomogram construction. Compared to these studies, our research incorporated a broader range of variables encompassing lifestyle factors, laboratory parameters, WC, visceral fat, and WHR, providing enhanced insights for predicting lean-NAFLD.

This study had some limitations that should be acknowledged. First, this was a single-center retrospective study that focused on an Asian population. Therefore, the applicability of this prediction model to other populations may be limited. Second, we did not assess the long-term outcomes of lean-NAFLD in these populations or the cost-effectiveness of the screening. The relationship between NAFLD and extrahepatic diseases should be investigated in the future. Third, there may be unknown confounding factors that could limit the accuracy of the LASSO model, such as genetic problems. Nevertheless, the development of this tool can aid in the early identification of at-risk NAFLD individuals. While the LASSO model developed in this study showed comparable predictive power to FLI, the use of machine learning for NAFLD prediction is a growing trend that allows for the inclusion of a broader range of predictive factors. In future studies, we plan to incorporate more variables to increase the accuracy of NAFLD prediction. Moreover, external validation using data from a second population or large-scale biobanks, such as the UK Biobank, would significantly strengthen our findings, will be a primary focus in our future research to enhance the robustness and applicability of our model.

## 5. Conclusion

We developed and validated a LASSO-derived prediction model based on four clinical parameters—visceral fat, triglyceride levels, HDL-C levels, and WHR. This model exhibited good performance in terms of predicting lean-NAFLD in an Asian population. Thus, we provide a personalized risk stratification screening strategy for NAFLD in these low-risk populations.

## Supplementary Material

Supplemental Material

## Data Availability

The study questionnaires and raw data are available from the project PI *via* email: cmvwu1029@gmail.com
