# Supplementary material for: LASSO-derived model for the prediction of lean-non-alcoholic fatty liver disease in examinees attending a routine health check-up
Source: Ann Med. 2024 Feb 16;56(1):2317348. doi: 10.1080/07853890.2024.2317348 (PMC10878349; doi:10.1080/07853890.2024.2317348)
Supplement: Supplemental Material [file IANN_A_2317348_SM7124.doc]

**Supplementary table 1**

Comparing the ability of FLI and LASSO model to predict lean-fatty liver when waist circumference was held constant. Assessing FLI and LASSO models' diagnostic performance in subgroups: males with waist circumference < 90 cm and females with waist circumference < 80 cm (N=918 subjects, delete male 36 subjects and female 208 subjects)

|  | **Cut off point** | **No fatty liver** | **Fatty liver** | **Accuracy** | **Sensitivity** | **Specificity** |
| --- | --- | --- | --- | --- | --- | --- |
| **LASSO model** | 1(≥0.1484) | 155 | 78 | 0.797 | 0.722 | 0.807 |
|  | 0(<0.1484) | 650 | 34 |  |  |  |
| **FLI** | 1 (≥15.45) | 159 | 76 | 0.790 | 0.691 | 0.803 |
|  | 0 (<15.45) | 649 | 34 |  |  |  |

Abbreviation: LASSO, least absolute shrinkage, and selection operator; FLI, fatty liver index
